# Atrophic Dermatofibroma: A Unique Dermatofibroma Variant

**DOI:** 10.7759/cureus.14570

**Published:** 2021-04-19

**Authors:** Nikolas Gutierrez, Antoanella Calame, Christof Erickson, Philip R Cohen

**Affiliations:** 1 General Practice, 1st Marine Division, 1st Combat Engineer Battalion, Camp Pendleton, USA; 2 Dermatology/Dermatopathology, Compass Dermatopathology, San Diego, USA; 3 Dermatology, Scripps Memorial Hospital, La Jolla, USA; 4 Dermatology, Compass Dermatopathology, San Diego, USA; 5 Dermatology, San Diego Family Dermatology, National City, USA

**Keywords:** acanthosis, atrophic, atrophy, dermatofibroma, elastic, fibers, fibroblast, hyperpigmentation

## Abstract

Dermatofibromas are benign skin tumors with several variants, including the rare, uncommonly described atrophic dermatofibroma. To the best of our knowledge, there are currently 105 reported cases of atrophic dermatofibromas in the literature. This variant typically presents as a flat or depressed macule whose color can range from brown to white to red; in contrast to classic dermatofibromas that typically occur on the legs, atrophic dermatofibromas have a tendency to occur on the upper back and arms. An atrophic dermatofibroma can be clinically diagnosed; however, given the broad spectrum of clinical features of this lesion, a biopsy may be required. Characteristic pathologic features include epidermal acanthosis, basilar hyperpigmentation, fibroblast hyperplasia, and decreased or absent elastic fibers within the lesion. The pathogenesis of this lesion is not yet fully understood; however, it has been postulated that the loss of elastic fibers plays a key role in its development and characteristic atrophic appearance. We present the cases of two men with biopsy-confirmed atrophic dermatofibromas: a 47-year-old man with a pigmented macule on the right upper back and a 68-year-old man with an erythematous patch on the left posterolateral shoulder. The clinical and pathologic features of atrophic dermatofibromas are also summarized.

## Introduction

Dermatofibromas are common, benign fibrohistiocytic lesions of the skin. There are numerous variants, including aneurysmal, atrophic, cellular, epithelioid, and several others. Specifically, atrophic dermatofibroma is a rare, uncommonly described variant [1,2].

Atrophic dermatofibroma was first coined by Page and Assad in 1987; however, there are reports of similarly described lesions in the French and Spanish literature that predates 1987. This specific variant has been observed worldwide with retrospective studies estimating its prevalence to be as low as 0.5-2% to as large as 13-18%. These lesions typically present as smooth, umbilicated, skin-colored, or erythematous macules that are often misidentified on initial clinical examination [2-4].

Two men with skin lesions on the right upper back or left posterolateral shoulder were observed. The diagnosis of atrophic dermatofibroma was suspected clinically and confirmed on histologic evaluation. The clinical and pathologic features of this unique dermatofibroma variant are summarized.

## Case presentation

Case 1

A 47-year-old man without any significant past medical history presented with a pigmented lesion on his right upper back that had been increasing in size during the prior month. The patient denied personal or family history of dermatologic conditions or malignancies.

**Figure 1:**
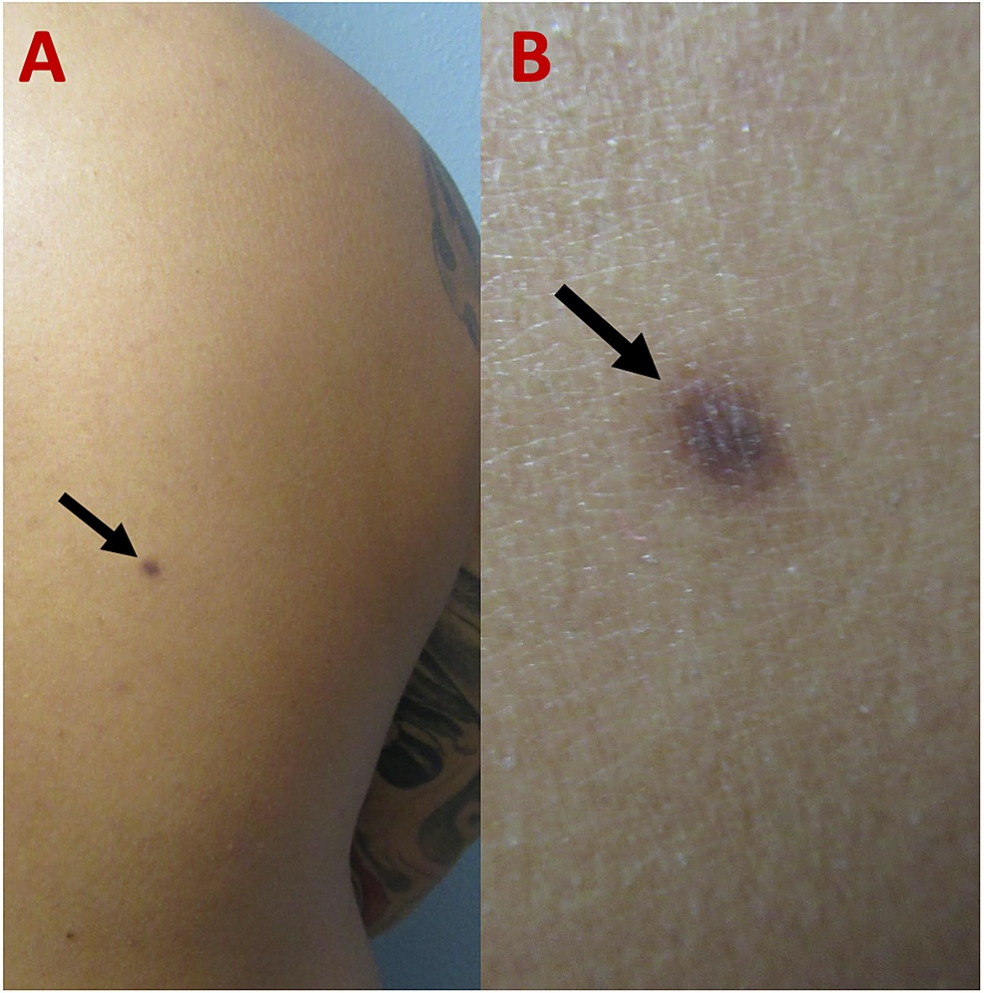
Clinical presentation of an atrophic dermatofibroma. Distant (A) and closer (B) views of a 47-year-old man with an atrophic dermatofibroma presenting as a flat, atrophic, hyperpigmented macule (black arrow) on his right upper back.

Cutaneous examination revealed a flat, atrophic, pigmented 5.0 × 5.0-mm macule on his right upper back (Figure 1). The initial clinical differential diagnosis included atrophic dermatofibroma, fibrous dermatofibroma, malignancy, nevus, and scar. A punch biopsy was obtained.

**Figure 2:**
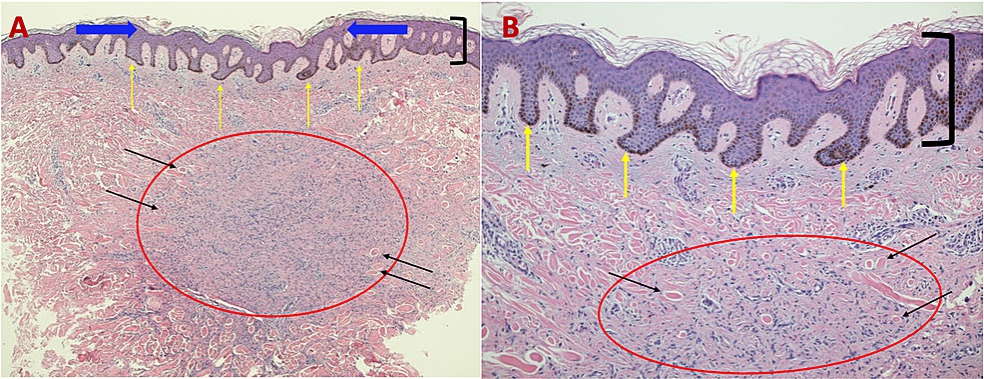
Microscopic presentation of H&E-stained sections of an atrophic dermatofibroma on the right upper back of a 47-year-old man. Low (A) and higher (B) magnification of H&E-stained sections of an atrophic dermatofibroma shows a central depression (between blue arrows), epidermal acanthosis (thickening of the epidermis as shown between black bracket), basilar hyperpigmentation (yellow arrows), and a fibroblast proliferation (red circle) with trapped collagen bundles at the periphery (black arrows) (H&E: A, ×5; B, ×20). H&E, hematoxylin and eosin

**Figure 3:**
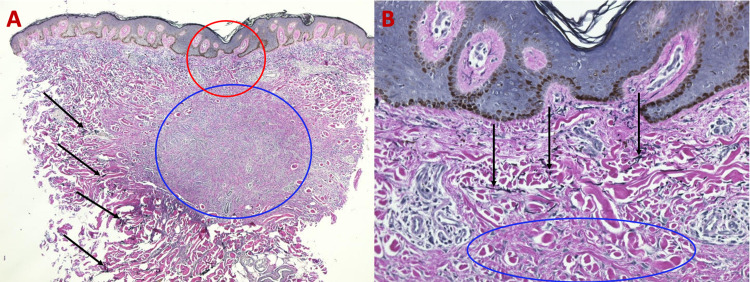
Microscopic presentation of VVG-stained sections of an atrophic dermatofibroma on the right upper back of a 47-year-old man. Low (A) and higher (B) magnification of VVG-stained sections of an atrophic dermatofibroma reveals a loss of black-stained elastic fibers (blue circle) within the lesion; in contrast, elastic fibers (black arrows) can be seen in the dermis above, adjacent, and deep to the dermal tumor. The red circle of image A is shown at higher magnification in image B (VVG: A, ×5; B, ×40). VVG, Verhoeff-van Gieson

Microscopic evaluation of the tissue revealed epidermal acanthosis, basilar hyperpigmentation, and fibroblast proliferation on hematoxylin and eosin (H&E) staining (Figure 2). Verhoeff-van Gieson (VVG) staining demonstrated a loss of elastic fibers in the lesion when compared to the adjacent dermis above, lateral, and below the tumor, which showed scant, black-stained elastic fibers (Figure 3).

Correlation of the clinical features and pathologic findings established the diagnosis of an atrophic dermatofibroma. The benign nature of this lesion was discussed with the patient, and no further treatment was performed. The residual lesion will continue to be monitored clinically.

Case 2

**Figure 4:**
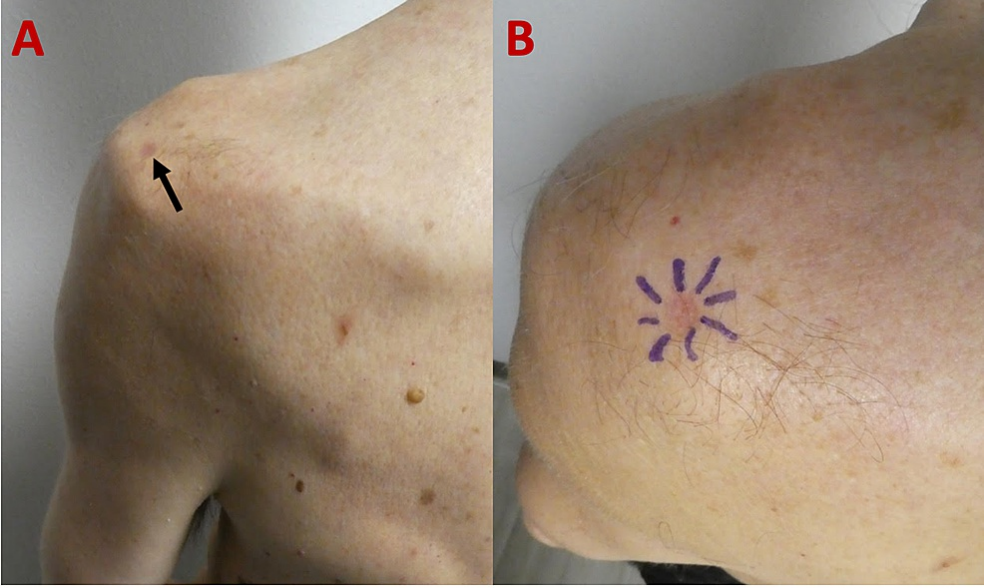
Atrophic dermatofibroma on the left posterolateral shoulder of a 68-year-old man. The distant (A) view of an atrophic dermatofibroma presenting as a depressed, erythematous patch (black arrow) on the left posterolateral shoulder. A closer view (B) demonstrates the peripheral edges of the central, atrophic tumor outlined by purple pen markings prior to biopsy specimen collection.

A 68-year-old man with no significant personal or family history of dermatologic conditions or malignancies presented for an evaluation of his skin. Cutaneous examination revealed a 6.0 × 5.0-mm depressed, erythematous patch on his left posterolateral shoulder (Figure 4). The clinical differential diagnosis included anetoderma, atrophic dermatofibroma, atrophoderma, dermatofibrosarcoma protuberans, and granuloma annulare. A punch biopsy was performed.

**Figure 5:**
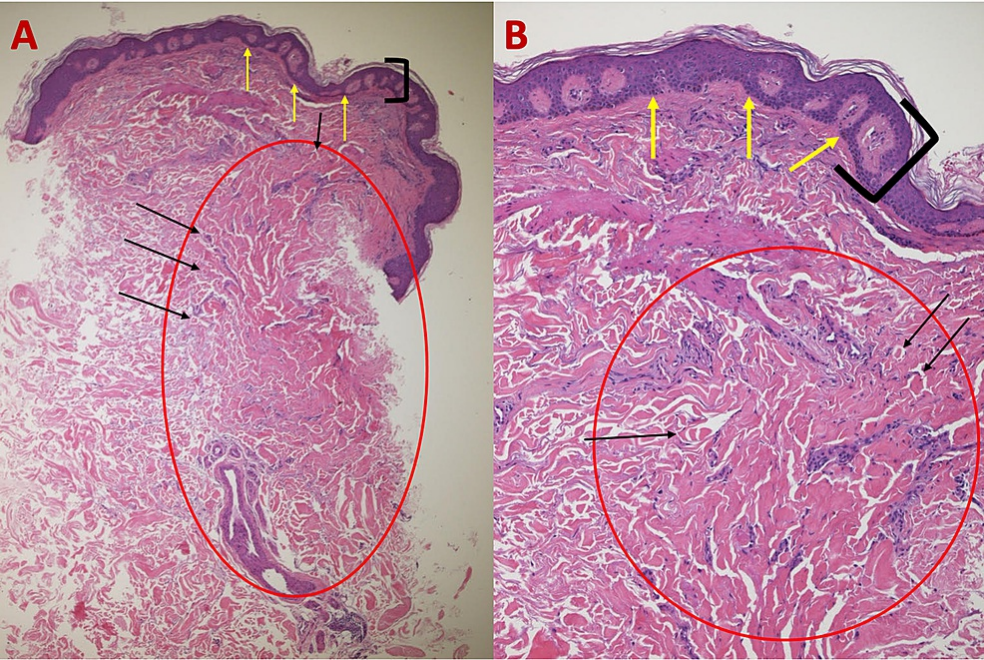
Histologic features of atrophic dermatofibroma on the left shoulder of a 68-year-old man on H&E sections. Low (A) and higher (B) magnification of H&E-stained sections of an atrophic dermatofibroma showing epidermal acanthosis (thickening of the epidermis as shown between black bracket), basilar hyperpigmentation (yellow arrows), and proliferation of dermal fibroblasts (red circle) with trapped collagen bundles (black arrows) (H&E: A, ×5; B, ×20). H&E, hematoxylin and eosin

**Figure 6:**
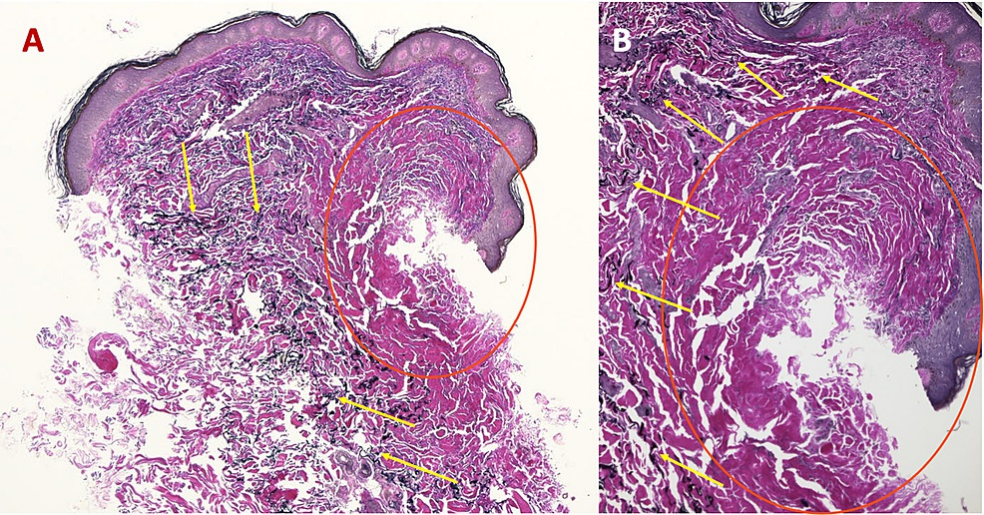
VVG-stained sections of an atrophic dermatofibroma on the left shoulder of a 68-year-old man. Low (A) and higher (B) magnification of VVG-stained sections of an atrophic dermatofibroma reveals an absence of elastic fibers within the tumor (red circle); however, black-stained elastic fibers (yellow arrows) can be visualized in the deep dermis above, adjacent, and deep to the dermatofibroma (VVG: A, ×5; B, ×20). VVG, Verheoff-van Gieson

Microscopic evaluation of the tissue specimen revealed epidermal acanthosis, heavy melanin deposits in the basilar layers of the epidermis, and focal fibroplasia in the dermis on H&E-stained sections (Figure 5). VVG-stained sections revealed an absence of elastic fibers within the lesion; however, they were present in the dermis above, adjacent to and below the lesion (Figure 6).

Correlation of the clinical and pathologic findings established the diagnosis of an atrophic dermatofibroma. The benign nature of the lesion was discussed, and no further treatment was performed. The residual lesion will continue to be monitored clinically.

## Discussion

Atrophic dermatofibroma is a less frequently described variant compared to the common fibrous dermatofibroma. To the best of our knowledge, this lesion has been reported in the literature in approximately 107 patients, including our two patients presented above. There were 55 women and 14 men, establishing a female-to-male ratio of 3.9 to 1.0. Gender was not disclosed for 38 individuals [2,5-7].

This rare fibrohistiocytic tumor was most commonly diagnosed in patients 40-49 years old; no known association between atrophic dermatofibroma and other cutaneous or systemic diseases has been identified. Unlike classic dermatofibromas that typically occur on the lower extremities, this rare variant is most common on the upper back and arms. Given its rarity and atypical presenting locations, it has been reported that the mean time from onset to diagnosis ranges between three and eight years [2,4,8].

Atrophic dermatofibroma is a benign tumor that typically presents as a flat, atrophic solitary lesion whose color can vary from flush white to red to brown. The size ranges from 5.0 × 7.0 mm to 5.0 × 5.0 cm. Typically, the lesion is asymptomatic; however, some patients have noted changes in size, associated pain, and intermittent swelling [2].

Dermoscopy has been reported as a possible tool to aid in proper clinical diagnosis of this rare variant. Dermoscopic findings of atrophic dermatofibroma include a patchy, peripheral pigment network with multiple white patches. With increased data collection and observation, dermoscopy may play an important role in accurate clinical identification of this uncommon tumor; however, because the clinical expertise of dermoscopy is dependent on the individual performing the evaluation, biopsy of a lesion that is morphologically suspected to be an atrophic dermatofibroma may be helpful to establish the diagnosis [4,9-11].

The differential diagnosis of atrophic dermatofibroma is broad and includes benign and malignant skin tumors such as amelanotic melanoma, atrophic dermatofibrosarcoma protuberans, basal cell carcinoma, dermatofibrosarcoma protuberans, desmoplastic melanoma, fibrous dermatofibroma, and squamous cell carcinoma. These benign and malignant neoplasms can morphologically appear similar to an atrophic dermatofibroma; key, nuanced features on gross clinical evaluation, dermoscopy, histology, and immunohistochemical staining may aid in accurate differentiation (Table 1). Other conditions that can also be considered in the clinical differential diagnosis of atrophic dermatofibroma include local dermatoses such as anetoderma, atrophoderma, non-specific granuloma, and panniculitis. Rarely, systemic illness such as lupus erythematous, morphea, and sarcoidosis have been suspected [1,2,9,10,12-20].

**Table 1:** Clinical differential diagnosis of atrophic dermatofibroma and key differentiating features. Ber-EP4, epithelial cell adhesion molecule; CD34, cluster of differentiation 34; D2-40, podoplanin; EMA, epithelial membrane antigen; factor XIIIA, blood coagulation prototransglutaminase; HMB-45, human melanoma black-45; IHC, immunohistochemistry; MART-1, melanoma antigen recognized by T-cells; p63, keratinocyte differentiation marker; S100, calcium binding protein; SOX10, sex-determining region Y-box 10; +, present; -, absent

Condition	Clinical presentation	Dermoscopy	Pathology	IHC staining
Amelanotic melanoma [1,12,13]	Skin-colored to red irregular tumor that may appear as a macule, plaque, or papulonodular form usually on the face or extremities	Erythematous to lightly pigmented lesion with irregular borders, peripheral pigment, and scattered red dots	May manifest as any histological melanoma subtype with amelanosis	-Factor XIIIa, -CD34, +S100, +HMB-45, +MART-1
Atrophic dermatofibroma [1,2,9,10]	Flat, atrophic macule or patch on upper back or extremities whose color can range from white to red to brown	Patchy, peripheral pigment network with multiple white patches	Loss of elastic fibers, dermal thinning, and central depression which may be greater than 50% in the locoregional dermis	+Factor XIIIa, -CD34, -S100, -HMB-45
Atrophic dermatofibromasarcoma protuberans [1,14,15]	Depressed, brown to red to gray plaque typically occurring on the trunk	Two reported cases: (1) Branching vessels with a yellowish background without pigment network. (2) Regular brown lines reticular on purplish-erythematous background	Ill-defined lesion with uniform spindle cells arranged in parallel or horizontally oriented fascicles and focal segments of storiform arrangements	-Factor XIIIa, +CD34, -S100, -HMB-45
Basal cell carcinoma [16,17]	Pearly, flesh-colored papule or erythematous macule with overlying telangiectasias in sun-damaged areas, most commonly the head and neck	Ulceration, multiple blue/gray lobules, maple leaf-like area, large blue/gray ovoid nests, spoke-wheel area, and/or arborizing telangiectasias	Nests of basaloid tumor cells with peripheral palisading and peritumoral retraction of the surrounding dermal stroma	-Factor XIIIa, -CD34, -S100, -HMB-45, +p63, -EMA, +Ber-EP4
Dermatofibrosarcoma protuberans [1,14,15,20]	Skin-colored or red, firm papule or plaque-like induration that progresses to a nodule with accompanied additional growths giving the characteristic protuberant appearance	Delicate pigment network, blood vessels, structureless light brown areas, shiny white streaks, pink background coloration, and structureless hypopigmented or depigmented areas	Ill-defined lesion with uniform spindle cells in storiform arrangement with minimal atypia	-Factor XIIIa, +CD34, -S100, -HMB-45, -D2-40,
Desmoplastic melanoma [18]	Non-specific amelanotic nodule or plaque typically on the head and neck, trunk, and extremities	White patch with linear-irregular vessels and/or milky-red areas, melanocytic structures (atypical globules), and atypical pigment network have also been reported	Pauci-cellular spindle-shaped melanocytes dispersed in abundant collagen matrix with deep infiltration, nerve involvement is common	-Factor XIIIa, -CD34, +S100, -HMB-45, -MART-1, +SOX10
Fibrous dermatofibroma [1,9,10,20]	Raised, cutaneous nodule with a red-brown surface with well-circumscribed borders on the extremities	Peripheral pigmented network with central scar-like white patch	Acanthosis of the epidermis, epidermal basal layer hyperpigmentation, and proliferation of spindle-shaped fibroblasts with cuboidal collagen bundles at the periphery of the lesion	+Factor XIIIa, +CD34, -S100, -HMB-45, +D2-40
Squamous cell carcinoma [17,19]	Red, scaly plaque or patch occurring most commonly on sun-damaged skin, may also present with ulceration in advanced disease	White-yellowish keratin mass with targetoid hair follicles, hairpin, and/or linear-irregular vessels may also be observed	Atypical keratinocytes that extend from the epidermis and infiltrate into the dermis	-Factor XIIIa, -CD34, -S100, -HMB-45, +p63, +EMA

Pathologic evaluation may be necessary to confirm the diagnosis of atrophic dermatofibroma as the differential diagnosis is broad. H&E staining of an atrophic dermatofibroma shares many histologic features with those of a classic fibrous dermatofibroma, including acanthosis of the epidermis, epidermal basal layer hyperpigmentation, and proliferation of spindle-shaped fibroblasts with cuboidal collagen bundles at the periphery of the lesion. Differentiating features of atrophic dermatofibroma are central depression, dermal thinning, and loss of elastic fibers [1,2,8,10].

Although a central depression can occasionally be observed in common dermatofibromas, early researchers have suggested that the central depression in atrophic dermatofibromas must be severe, measuring greater than 50% in the locoregional dermis. The central depression and atrophic appearance of the lesion have been associated with the decrease or complete loss of elastic fibers in the lesion with a normal distribution of elastic fibers at the periphery; this is most readily seen on VVG-stained sections. Both of our patients had a significant decrease in elastic fibers within their lesions with normal-appearing distribution of elastic fibers in the dermis above, adjacent to and beneath the atrophic dermatofibroma [1,2,8,10,11].

Immunohistochemical staining of a biopsy tissue specimen may aid in identification of an atrophic dermatofibroma, ensuring proper diagnosis, treatment, and prognosis. Atrophic dermatofibromas characteristically stain positive for factor XIIIa and negative for cluster of differentiation 34, human melanoma black-45, and S100, differentiating this benign lesion from similarly presenting malignant neoplasms such as amelanotic melanoma, dermatofibrosarcoma protuberans, and desmoplastic melanoma. A novel immunohistochemical stain, podoplanin (D2-40), is emerging as another marker in differentiating between dermatofibroma and dermatofibrosarcoma protuberans; however, testing on the atrophic variants of these lesions has not yet been completed (Table 1) [1,2,9,10,12-20].

The pathogenesis of atrophic dermatofibroma remains to be elucidated. However, it has been postulated that the loss of elastic fibers plays an integral role in the development of the lesion and its dimpled, atrophic appearance. Furthermore, it has been reported that the paucity of elastic fibers may be due to elastophagocytosis by dermatofibroma cells and/or elastolysis caused by overexpression of matrix metalloproteinase-1 in the tumor cells [2,6,11].

Another postulated mechanism for the atrophic clinical appearance of this lesion may be related to the subcutaneous tissue atrophy present beneath the lesion. However, deep tissue samples are not typically included in the submitted biopsy specimen; therefore, observation to confirm this is limited. Hence, loss of elastic fibers or subcutaneous tissue biopsy or both may have a role in the development of atrophic dermatofibroma [2,6,11].

Atrophic dermatofibromas are benign lesions. If clinically diagnosed, no further intervention is necessary. If the diagnosed is established histologically and a portion of the tumor has been removed, it is reasonable to periodically monitor the residual lesion [2].

## Conclusions

Atrophic dermatofibroma is an uncommon, benign variant of a dermatofibroma. There are 107 cases described, and the tumor occurs almost four times more often in women than men. Moreover, we suspect that the prevalence of this lesion is greater than the number of reported cases in the literature as the lesion may be misdiagnosed because of its atypical presentation. Atrophic dermatofibroma typically presents as an erythematous or hypopigmented, atrophic macule on the upper back and arms. The clinical differential diagnosis of atrophic dermatofibroma is broad and includes benign and malignant neoplasms, local dermatoses, and systemic conditions. The diagnosis can be suspected clinically based on clinical features and confirmed histologically with microscopic examination of a tissue specimen. Characteristic histologic findings include the loss of elastic fibers, dermal thinning, and central depression which may be greater than 50% in the locoregional dermis. In our patients, atrophic dermatofibroma was clinically suspected and confirmed on evaluation of the biopsy tissue specimen. Because an atrophic dermatofibroma is benign, excision is not required if the tumor is clinically diagnosed. Periodic monitoring of the residual lesion can be considered if a partial biopsy of the lesion was performed.
